# MOTEMO-OUTDOOR: ensuring learning and health security during the COVID-19 pandemic through outdoor and online environments in higher education

**DOI:** 10.1007/s10984-023-09456-y

**Published:** 2023-02-09

**Authors:** Corel Mateo-Canedo, Neus Crespo-Puig, Ramon Cladellas, Jorge Luis Méndez-Ulrich, Antoni Sanz

**Affiliations:** 1 Department of Basic, Developmental and Educational Psychology, Universitat Autonoma de Barcelona, Carrer de la Fortuna, S/N. Bellaterra Campus, 08193 Cerdanyola del Vallès, Barcelona, Spain; 2 Department of Methods of Research and Diagnosis in Education, Universitat de Barcelona, Barcelona, Spain

**Keywords:** e-learning, Face-to-face learning, Higher education, Informal learning, Outdoor learning, Pandemic, Student engagement

## Abstract

The restriction measures put in place during the COVID-19 pandemic posed notable challenges for formal teaching–learning processes because they had to be adapted to ensure health security. An active learning programme applied to three environments (indoors, outdoors, and online) was tested with 273 undergraduate university students in a within-subjects experimental study. Each student was assigned to two indoor and two outdoor seminars, with a subsample (*n* = 30) also participating in online seminars implemented in response to the university's lockdown protocols. The learning experience and learning conditions were evaluated through six dimensions: learning, evaluative impact, hedonic experience, technical conditions, environmental conditions, and health security. Outdoor seminars were more effective than indoor seminars in terms of the learning experience, with greater differences in hedonic experience, while the indoor seminars were rated more highly than the outdoor seminars in terms of learning conditions, with a larger difference in the environmental conditions. No differences were found between online and face-to-face environments in terms of the learning experience, even though the online environment yielded better scores in the learning conditions. Apparently, this adaptation to both outdoor and online contexts through active methodologies allows overcoming of technical, environmental, and teaching limitations and improves health security, while ensuring a good learning experience and added flexibility to teaching–learning processes.

**Supplementary Information:**

The online version contains supplementary material available at 10.1007/s10984-023-09456-y.

## Introduction

COVID-19 has not only had a high impact on public health (WHO, 2020, 2021), but associated restriction measures have also caused sudden and abrupt changes in the daily life of the population and challenged their well-being (Brooks et al., 2020). Management of the pandemic affected people’s routines from the first wave because of strict lockdowns, curfews, and closing of businesses (Eurofound, 2020), as well as academic life (Aristovnik et al., 2020; European Commission, 2021; OECD, 2020). Teachers were forced to suddenly adapt their strategies and methods to an online modality (Bao, 2020; Bozkurt, & Sharma, 2020; Maile et al., 2020). The reincorporation into university classrooms occurred in many countries in a staggered manner, with a return to online classes for some periods and with many social distancing restrictions remaining in place even months after the end of the first wave of the pandemic to avoid spreading the SARS-CoV-2 virus. Some experiences such as the HyFlex model adopted by King's College (Detyna et al., 2022) were implemented to overcome this problem by combining face-to-face with synchronous online contexts. This shift revealed several challenges related to student, environmental and external limitations, the student experience, and academic and safety requirements. As a result of the scientific evidence suggesting that SARS-CoV-2 is mainly transmitted through the air, it was also proposed to adopt measures very different from those mentioned above, such as outdoor learning (Melnik & Darling-Hammond, 2020), in the return to academic activity after the confinement period, thus adding protection against the transmission of the coronavirus to the intrinsic benefits of using outdoor spaces for learning (Quay et al., 2020). These proposals have their precedent in the introduction of outdoor learning to deal with the 1918–1919 influenza pandemic (Afshar & Barrie, 2020) and have been part of the policies implemented by some governments (e.g. Denmark) during the COVID-19 pandemic (Sheikh et al., 2020). Experiences prior to the pandemic had shown the benefits of outdoor education for aspects such as the well-being, mental health, and physical, socio-emotional, and cognitive development of students, as well as higher academic performance among students in various subjects such as mathematics, language, arts, sciences, and social studies (Parker, 2022). In parallel, there seems to be a clear improvement in the self-perception of students in these skills (Thomas, 2018) while also enhancing skills such as resilience, collaboration, conflict resolution, and self-regulation (Mann et al., 2021).

Learning can occur in a variety of environments (formal and informal). The characteristics of a learning environment can have a significant impact on student learning and the use of certain methodologies supported by digital technology (He & Li, 2019). Recent years have seen university professors become progressively more interested in informal learning, re-thinking their approach and using other methods that differ from more traditional methods such as master classes or face-to-face practices (Carrillo & Flores, 2020). This interest even increased during the COVID-19 pandemic, which has served as a catalyst for previous initiatives in relation to a change of teaching and learning strategies (Hargreaves, 2021) to overcome the impact of isolated and online teaching on the student learning experience (Ali, 2020; Kavaric et al., 2021). The pandemic has accelerated interest in promoting student engagement and motivation in online teaching (Best & MacGregor, 2017; Muir et al., 2019). During the lockdown period, the so-called active learning methods were popular for promoting and enhancing the participation and engagement of students. These teaching methods include self-managed learning groups (Lizzio & Wilson, 2005), online case studies (Luo et al., 2018), flipped online classrooms (Dooly & Sadler, 2020) and gamification (Subhash & Cudney, 2018) among many others. In addition, the main way to manage the classes involved using information and communication technologies (ICT), which have a high potential to promote active and meaningful learning (Ferdig et al., 2020). Several studies conducted before the pandemic reported the benefits of using social networks, such as Facebook® and smartphone apps, as educational vehicles (Aydin, 2012; Fu, 2013; Jesse, 2015), or even more disruptive and ‘out of the box’ strategies such as podcasting (Forbes & Khoo, 2015). These ICT-based learning activities should be developed because high arousal positive emotions—as well as high or low arousal negative valence emotions—have a considerable impact on the learning process (Racanello et al., 2022). Studies conducted on the forced adaptation of learning processes to the online environment because of the pandemic have yielded mixed results. Thus, there seems to be a consensus among different students and educational staff about the opportunities offered by the online environment to ensure the continuity, usability, and efficiency of learning processes (Kurbakova et al., 2020. However, students have also revealed a certain degree of scepticism concerning their training in technical and practical skills but not in the acquisition of abstract knowledge (Abassi et al., 2020), along with a perceived loss of motivation and learning in the transition from the synchronous face-to-face environment to the online environment (Tan, 2020). In the opinion of students and teaching staff, the success of implementing virtual environments during the pandemic mainly depended on the efforts to adapt practical classes to such an environment, the extent to which the material was adequately structured, the availability of technical support to combat connection difficulties, and whether a good student–teacher interaction could be guaranteed (Nambiar, 2020). In any case, there is a consensus that the COVID-19 pandemic has been perceived as an opportunity to introduce online learning (Pokhrel & Chhetri, 2021).

Therefore, the health crisis has represented both a challenge and a test for the structure of the university system regarding the possibilities of being able to maintain academic activities that preserve as much as possible the quality of teaching and learning while protecting the health of individuals (Czerniewicz, 2020; Faura-Martínez et al., 2021; Goutam et al., 2021). The COVID-19 pandemic has emphasised the relevance of the social and participatory dimension of education, transcending the vision of students as simple users of a service and placing them as key agents involved in their learning. This is why, despite the contribution that online teaching made to the maintenance of teaching during the pandemic, there was a need to find ways to resume face-to-face educational activities while maintaining security measures against COVID-19.

In a context of the progressive recovery of face-to-face interaction in higher education amid the pandemic, we designed and implemented a teaching innovation experience called MOTEMO-OUTDOOR to protect health while carrying out academic activities. This program was designed to take advantage of the fact that our university has a campus with many accessible and easily adaptable green spaces near the classrooms. Thus, aside from the health protection afforded by open spaces, we were also able to exploit the positive effects on cognitive processes of exposure to natural environments (Kaplan, 1995), making safe learning not only possible but even enhancing it. The feasibility and effectiveness of outdoor active learning methodology was tested and compared with regular face-to-face indoor classroom discussion seminars. An online asynchronous adaptation was also designed for use if government decisions to manage the pandemic forced a return to lockdown, thus providing flexibility in the face of such uncertainty. The research questions in this study were:

1. Is it possible to design and implement learning activities in informal learning environments (outdoor and online) while ensuring the same quality of learning experiences as those provided in the classroom?
2. What are the differences in the learning environment provided by these three contexts or formats of teaching–learning procedures?
3. How does the learning environment relate to the learning experience?

Despite appeals for the adoption of measures such as outdoor learning, the scientific evidence on the implementation and impact of this type of experience is scarce and is particularly focused on the first education stage (i.e., kindergarten), in the context of so-called *outdoor education* (Spiteri, 2020). In fact, to our knowledge, this is the first reported experience of a teaching innovation in higher education employing an outdoor environment during the COVID-19 pandemic that has been accompanied by an empirical study of implementation and impact analysis. This experience is doubly innovative because it is combined with the implementation of a complementary adaptation to online learning.

## Method

### Participants

**Fig. 1:**
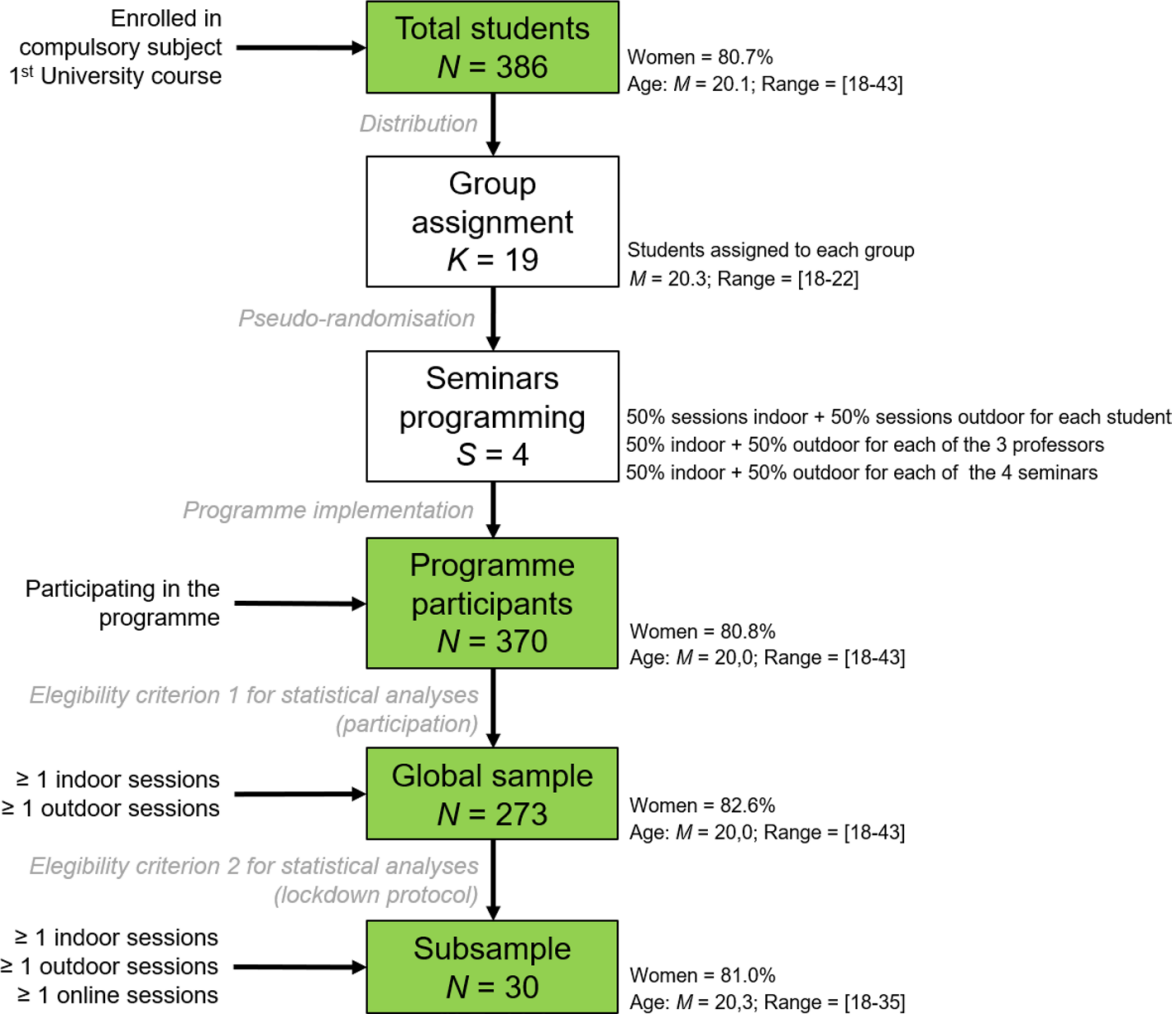
Sociodemographic characteristics of the participants in the four stages of the study

A total of 386 students in the first year of a psychology degree at the Autonomous University of Barcelona and enrolled in the compulsory subject Psychological Processes: Motivation and Emotion (abbreviated MOTEMO) were invited to participate in the MOTEMO-OUTDOOR programme as part of their formal training. A total of 370 students (95.8%) participated in at least one of the four scheduled sessions of the seminars (Fig. 1). Of these, 273 students met the eligibility criterion 1 consisting of having participated in at least one indoor and one outdoor session; this was the final global sample analysed in the study. Of the global sample, 78.9% were women and the mean age was 20 years (range 18–43, 90.4% of students were between 18 and 20 years old). A total of 30 students also met the eligibility criterion 2 consisting of having participated in at least one seminar session held in an asynchronous virtual environment because of COVID-19 health protocols that were in place at the university at that time; this constituted the subsample for specific analysis.

### Material and instruments

An impact assessment questionnaire made up of a total of 6 items was developed in the Catalan. Three of the items that evaluate the learning experience of each seminar were derived from a gamification programme (Martínez-Guillamón et al., 2017), whereas three items for evaluating the environmental conditions of each seminar were developed ad-hoc for the proposals of this study. Each of the items evaluates one of the dimensions of interest for the study: (1) degree of learning (“Indicate the degree of learning that you think this seminar has provided you with”); (2) evaluative impact (“Indicate to what extent you think this seminar contributes to improving your grade in the subject”); (3) hedonic experience (“indicate the degree of fun/positive emotions evoked by this seminar”); (4) technical conditions (“Indicate the degree to which the technical conditions—Internet connection, access to the material—have allowed you to carry out the activity”); (5) environmental conditions (“Indicate to what extent the environmental conditions—lighting, noise, temperature, etc.—have allowed you to carry out the activity”); and (6) health safety (“Indicate to what extent you felt safe during the activity in relation to COVID-19”). Each of the items was measured on an 11-point scale (0–10) accompanied by ‘minimum’ and ‘maximum’ labels next to the extreme values of the scale. The form gathered sociodemographic data (age and gender), along with information on which teacher delivered the session and whether the format was indoor (synchronous), outdoor (synchronous) or online (non-synchronous). The questionnaire was designed to be administered on any of the electronic devices used by students (smartphone, tablet, or laptop). It was included as a section at the end of the self-administered form used as didactic material to enhance the dynamics of the seminar sessions.

### Study design and procedure

**Fig. 2:**
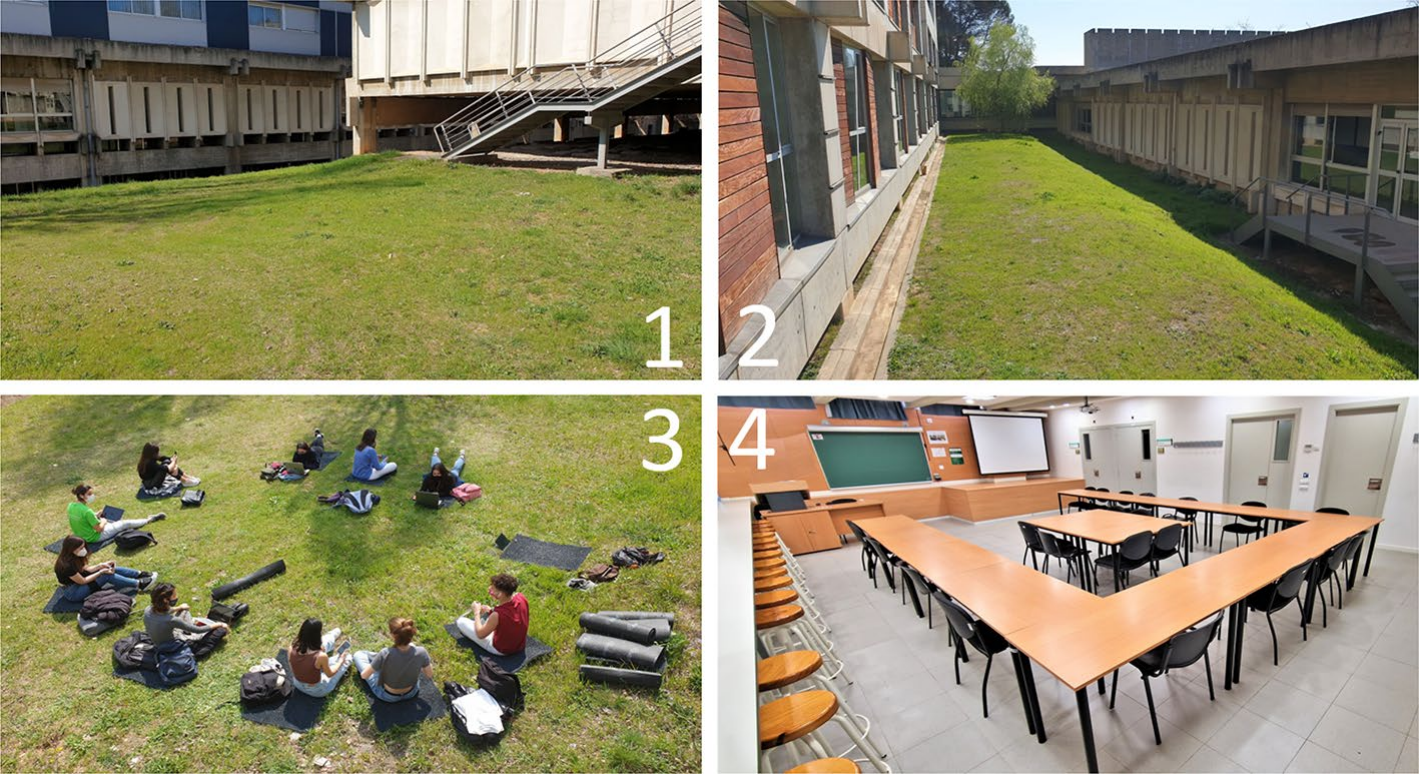
Spaces and settings of outdoor seminars. A1. Space “A”, geographical coordinates MOTEMO-OUTDOOR. A2: Seminar carried out in space “A”. B1:A1. Space “E”, geographical coordinates X XXº XX.XXX; X 2º X.XXX [blinded]. B2: Seminar carried out in space “E”

This was a quasi-experimental study with a within-subject design, in which each student participated in two conditions (indoor and outdoor) for the seminars for the subject (see Fig. 2 and supplementary material SM1 and SM2 for a detailed description of the didactic and logistic adaptations for the outdoor format). The study was carried out across a total of 76 seminars scheduled in the subject X [blinded] during the 2020–21 academic year between the months of March and June. Students were assigned to the indoor or outdoor seminar classes based on the pre-existing organisation of the subject, with students distributed in alphabetical order between 19 subgroups of *n* = 20 ± 2 students (see Supplementary Material SM3). Students attended the seminars in weeks 08/03/21 and 22/03/21 (S1), 26/03/21 and 09/04/21 (S2), 17/05/21 and 31/05/21 (S3), 31/05/21 and 04/06/21 (S4) according to the timetabling of the subject. Pseudo-randomisation of the context (indoor vs. outdoor) assigned 50% of sessions per group of students and per teacher. Likewise, the order of completion by student and teacher was counterbalanced (see Supplementary Material SM4). A total of 38 outdoor sessions were held, with a distribution of 50% for each student and close to 50% for each teacher and seminar. The impact questionnaire was completed by students immediately after each seminar in which they participated.

### Data preparation

Statistical analyses were conducted using IBM-SPSS® v.28. A product-moment Pearson correlation matrix was created for the three teaching formats (see Supplementary Materials SM5, SM6, and SM7) to reveal generalised intercorrelations between measures of the six dimensions evaluated. Therefore, the data were reduced to simplify the statistical treatment of the 24 impact indicators (6 variables × 4 evaluation time points) which consisted of (1) exploratory factor analysis for the reduction of dimensions and (2) calculation of the mean score of each participant in the three learning formats (indoor face-to-face, outdoor face-to-face, and asynchronous virtual).

Factor analysis (maximum likelihood) was conducted to identify possible higher-order factors in the six dimensions evaluated, including the 24 items resulting from the six indicators evaluated across the four seminars. The sedimentation plot clearly identified two high-order factors with an eigenvalue greater than 2, which jointly explained 45.3% of the variance. Oblimin rotation identified two factors consisting of 12 items with factor loadings > 0.4. Factor 1 explained 26.2% of the variance with three items from the four seminars related to the improvement in learning, the improvement in qualifications and the hedonic experience. Factor 2 explained 19.1% of the variance with three items from the four seminars related to environmental conditions, technical conditions, and health security. These factors were named *learning experience* and *learning conditions*, respectively. Cronbach's alpha internal consistency index revealed optimal reliability for both the learning experience scale (*α* = 0.91) and the learning conditions scale (*α* = 0.83). For all participants, the calculation was made by summing the learning experience and the learning conditions scores for each of the three learning formats (indoor face-to-face, outdoor face-to-face, and asynchronous virtual). A linear analysis of variance model was carried out with the total sample, with a two-level within-subjects factor (outdoor vs. indoor) for each of the dimensions evaluated and for the higher-order factors derived from the factor analysis. A linear analysis of variance model was also carried out with the subsample of 30 participants with a three-level within-subjects factor (indoor *vs*. outdoor *vs*. online). In this case, Bonferroni post-hoc contrasts were used to compare online with both face-to-face environments.

## Results

### Comparison of face-to-face contexts (indoor vs. outdoor)

**Fig. 3:**
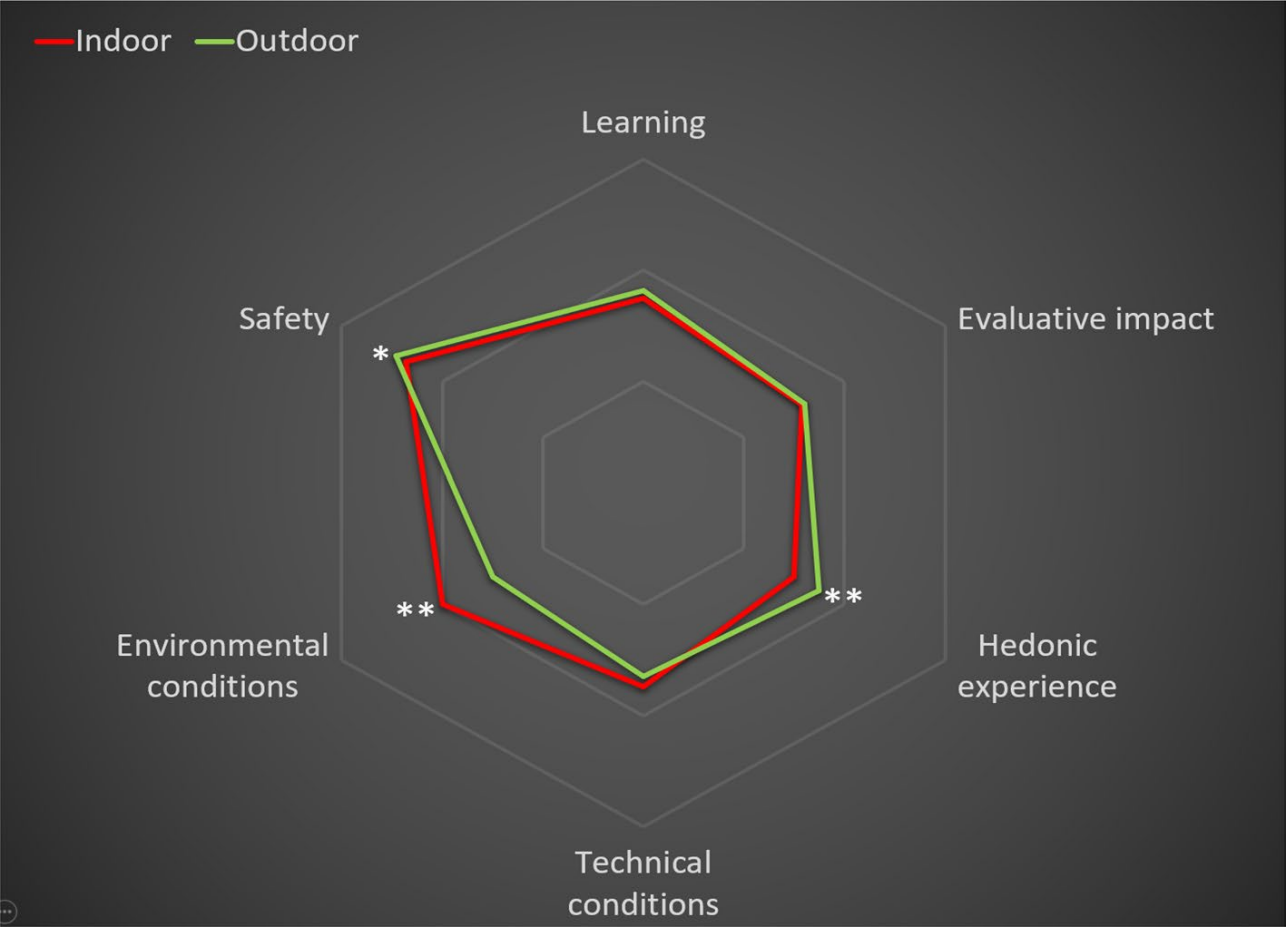
Graph showing means for the six dimensions evaluated for indoor versus outdoor conditions for the total sample that met with eligibility criteria (*N* = 273). *p < .05; **p < .001

Concerning the overall sample (Table 1 and Fig. 3), the linear analysis of variance model revealed a statistically significant difference between the two face-to-face contexts in relation to the learning experience high-order factor, with the mean score for this indicator being higher for the outdoor context (*M*_indoor_ = 25.82; *M*_outdoor_ = 26.18; *F*_(1, 272)_ = 4.28; *p* = 0.03; *η*_*p*_^2^ = 0.02). The dimensional analysis indicated that this was associated with an absence of differences in learning (*M*_indoor_ = 8.73; *M*_outdoor_ = 8.83; *F*_(1, 272)_ = 2.25; *p* = *0.13*; *η*_*p*_^2^ = 0.01) and evaluative impact (*M*_indoor_ = 8.58; *M*_outdoor_ = 8.60; *F*_(1, 272)_ = 0.06, p = 0.80; *η*_*p*_^2^ = 0.00), while the hedonic experience was evaluated as slightly higher in the outdoor context (*M*_indoor_ = 8.51; *M*_outdoor_ = 8.75; *F*_(1, 272)_ = 7.65, *p* = *0.006*; *η*_*p*_^2^ = 0.03).

**Table 1:** General linear model applied to six dimensions and two higher-order factors for comparing indoor vs outdoor conditions for total sample (*N* = 273)

High-order factor	Dimension	Indoor		Outdoor		ANOVA		
		*M*	*SD*	*M*	*SD*	*F*	*P*	*ɳ*_*p*_^*2*^
Learning experience	Total	25.82	3.06	26.18	2.93	4.28	.03*	.02
	Learning	8.73	1.08	8.83	1.08	2.25	.13	.01
	Evaluative impact	8.58	1.19	8.60	1.16	.06	.80	.00
	Hedonic experience	8.51	1.29	8.75	1.29	7.65	.006*	.03
Learning conditions	Total	27.17	3.16	26.64	2.98	6.97	.009*	.03
	Technical conditions	8.75	1.64	8.65	1.57	.75	.39	.00
	Environmental conditions	9.07	1.38	8.52	1.60	20.82	< .001**	.07
	Health safety	9.36	0.98	9.47	0.84	3.70	.05*	.01

*M* = Mean; *SD* = standard deviation; *ɳ*_*p*_^*2*^ = partial eta-square; *p < .05; ** *p* < .001

Moreover, this analysis indicated a statistically significant difference between the two face-to-face contexts in relation to the learning conditions, with the score for this factor being higher in the indoor context (*M*_indoor_ = 27.17, *M*_outdoor_ = 26.64, *F*_(1, 272)_ = 6.97, *p* = 0.009, *η*_*p*_^2^ = 0.03). The analysis of dimensions indicated a more positive evaluation of environmental conditions in the indoor context (*M*_indoor_ = 9.07; *M*_outdoor_ = 8.52; *F*_(1, 272)_ = 20.82; *p* < *0.001*; *η*_*p*_^2^ = 0.07) and no difference in relation to the technical conditions (*M*_indoor_ = 8.75; *M*_outdoor_ = 8.65; *F*_(1, 272)_ = 0.75, *p* = *0.39*; *η*_*p*_^2^ = 0.00), while perceived safety in relation to COVID-19 was higher in the outdoor setting (*M*_indoor_ = 9.36; *M*_outdoor_ = 9.47; *F*_(1, 272)_ = 3.70, *p* = *0.05*.; *η*_*p*_^2^ = 0.01).

### Moderating factors

The variables identified as moderators (gender, age, and teacher) or confounders (number of sessions, order of seminar context, and type of seminar) were subjected to an analysis of variance as factors that could explain the variability in the outcomes evaluated. Gender appeared to be the only moderator that explained variance in the learning experience and learning conditions factors. Analysis of variance (Table 2) revealed a significant gender x context interaction in relation to the learning experience, with men indicating a more-positive learning experience than women in the indoor context (*M*_men_ = 26.37 vs. *M*_women_ = 25.74) and women indicating a more-positive learning experience than men in the outdoor context (M_men_ = 25.55 vs. *M*_women_ = 26.31; *F*_(1, 271)_ = 8.31,* p* = 0.004; *η*_*p*_^*2*^ = 0.03). This difference was also present in the two constitutive dimensions of learning and hedonic experience, but not in the impact on evaluation. Regarding the learning conditions, no statistically significant differences were found for gender or for the context x gender interaction. However, dimensional analysis revealed significant differences in perceived health security, with men scoring higher than women (*M*_(men)_ = 9.64 vs. *M*_(women)_ = 9.31; *F*_(1,271)_ = 3.70, *p* = 0.04, *η*_*p*_^*2*^ = 0.02). Finally, men tended to have a lower score than women on technical conditions and environmental conditions, although these differences did not reach statistical significance.

**Table 2:** General linear model applied to the six dimensions and two higher-order factors as a function of gender and context (indoor vs outdoor) for total sample (*N* = 273; bolded p < .05)

High-order factor	Dimension	Gender	Indoor		Outdoor		Gender			Format			Gender x Format		
			*M*	*SD*	*M*	*SD*	*F*	*P*	*ɳ*_*p*_^*2*^	*λ*	*p*	*ɳ*_*p*_^*2*^	*λ*	*p*	*ɳ*_*p*_^*2*^
Learning experience	Total	Men	26.37	2.87	25.55	3.36	.02	.88	.00	2.64	.61	.00	8.31	.004*	.03
		Women	25.74	3.08	26.31	2.82									
	Learning	Men	8.98	0.96	8.63	1.34	.03	.87	.00	.74	.39	.00	7.76	.006*	.03
		Women	8.69	1.10	8.87	1.02									
	Evaluative impact	Men	8.71	1.08	8.46	1.26	.01	.92	.00	.77	.38	.00	2.31	.13	.01
		Women	8.57	1.21	8.63	1.14									
	Hedonic experience	Men	8.69	1.31	8.45	1.40	.17	.68	.00	.14	.71	.00	5.37	.02*	.02
		Women	8.48	1.29	8.81	1.27									
Learning conditions	Total	Men	27.12	4.13	26.05	3.58	.70	.40	.00	4.81	.008*	.03	1.23	.27	.01
		Women	27.17	2.97	26.73	2.85									
	Technical conditions	Men	8.62	2.14	8.21	1.89	2.13	.14	.01	2.25	.14	.01	1.59	.21	.01
		Women	8.76	1.55	8.73	1.50									
	Environmental conditions	Men	8.86	2.08	8.22	1.83	2.43	.12	.01	11.78	.001*	.04	.09	.76	.00
		Women	9.10	1.22	8.56	1.56									
	Health safety	Men	9.64	0.77	9.62	0.83	3.70	.04*	.02	.49	.48	.00	.93	.34	.00
		Women	9.31	1.01	9.44	0.85									

*M* = Mean; *SD* = standard deviation; *λ* = Wilk’s Lambda*; ɳ*_*p*_^*2*^ = partial eta-squared; *p < .05; ** *p* < .001

Considering the interrelationships between the dimensions of learning experience and learning conditions, an analysis of covariance of the learning experience according to gender was carried out, with the adjustment variables of technical conditions, environmental conditions, and safety. In the case of the outdoor context, adjusting this covariate eliminated the significant effect of gender on the learning experience (absence of covariates: *F*_(1,309)_ = 4.42; *p* = 0.03; introducing covariates: *F*_(1,309)_ = 3.31; *p* = 0.07).

### Comparison of synchronous face-to-face vs. online environments

**Fig. 4:**
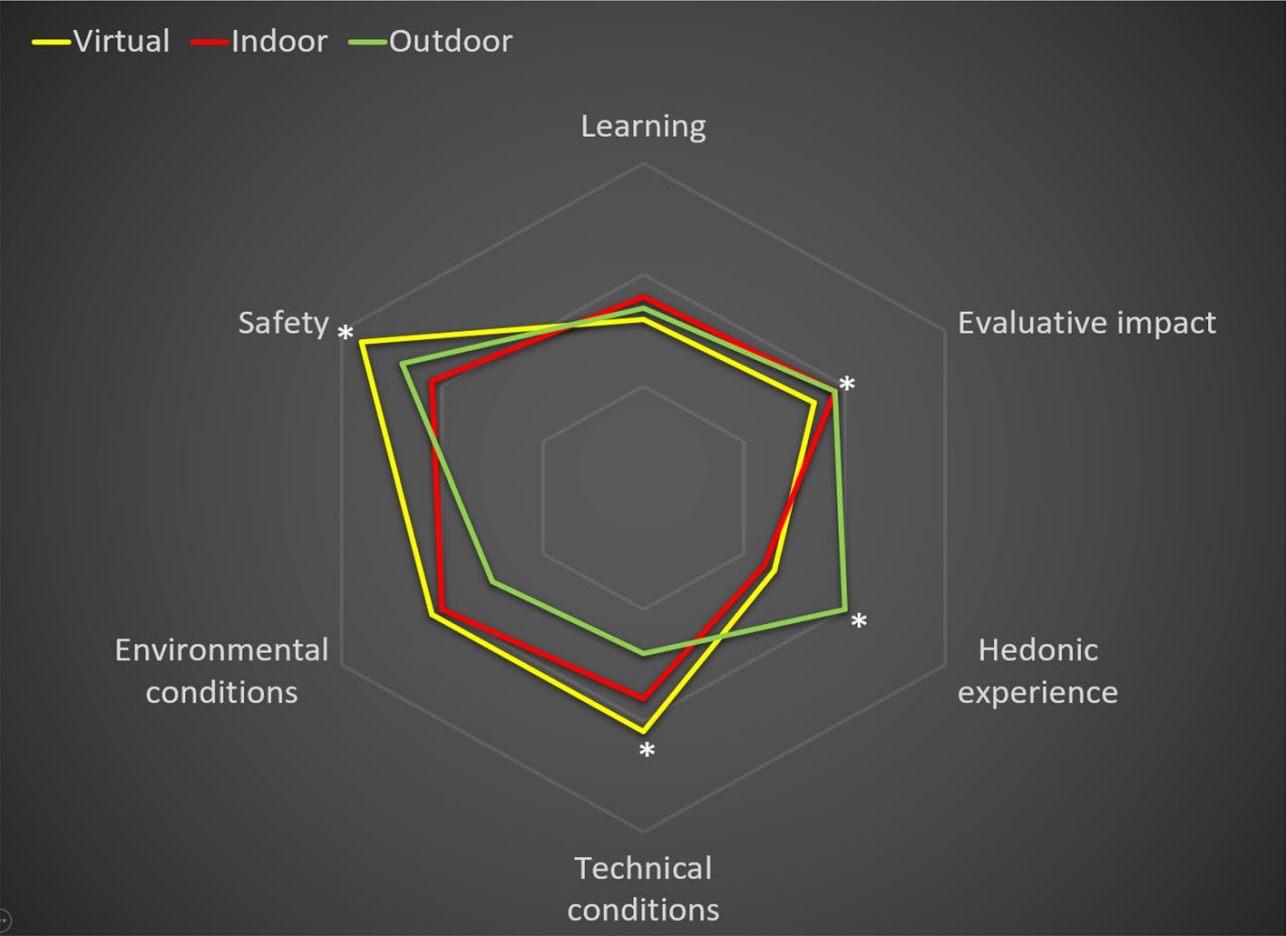
Graph showing means for the six dimensions evaluated for virtual vs indoor vs outdoor conditions for the subsample (*n* = 30). *p < .05; **p < .001

Regarding the subsample (*n* = 30; see Table 3 and Fig. 4), linear analysis of variance revealed statistically significant differences between the learning contexts in hedonic experience (*M*_*outdoor*_ = 9.02; *M*_*indoor*_ = 8.18; *M*_*virtual*_ = 8.25; *F*_(1, 28)_ = 5.22, *p* = 0.01; *η*_*p*_^*2*^ = 0.27). Bonferroni post-hoc tests revealed a significant difference in this dimension with a large effect size, with hedonic experience being greater in the outdoor context compared with the virtual context (*M*_*outdoor*_ = 9.02; *M*_*virtual*_ = 8.25; *F*_(1, 28)_ = 8.20, *p* = 0.008; *η*_*p*_^*2*^ = 0.22).

**Table 3:** General linear model applied to six dimensions and two higher-order factors comparing virtual vs indoor vs outdoor conditions for reduced sample (*n* = 30; bolded p < .05)

High-order factor	Dimension	Virtual		Indoor		Outdoor		ANOVA			Bonferroni post-hoc contrasts					
											Virtual vs Indoor			Virtual vs Outdoor		
		*M*	*SD*	*M*	*SD*	*M*	*SD*	*F*	*P*	*ɳ*_*p*_^*2*^	*F*	*p*	*ɳ*_*p*_^*2*^	*F*	*p*	*ɳ*_*p*_^*2*^
Learning experience	Total	25.57	4.01	25.83	3.57	26.58	3.29	1.49	.24	.10	.34	.56	.01	3.08	.08	.09
	Learning	8.63	1.37	8.78	1.20	8.72	1.24	.65	.53	.04	1.26	.27	.04	.15	.70	.00
	Evaluative impact	8.68	1.35	8.87	1.06	8.85	1.20	.53	.59	.04	1.02	.32	.03	.63	.43	.02
	Hedonic experience	8.25	1.56	8.18	1.60	9.02	1.14	5.22	.01*	.27	0.07	.79	.00	8.20	.008*	.22
Learning conditions	Total	27.95	2.80	27.00	3.82	26.35	3.25	3.81	.03*	.21	2.59	.11	.08	6.52	.01*	.18
	Technical conditions	9.13	1.41	8.83	1.73	8.37	1.80	2.51	.09	.15	.77	.39	.03	5.20	.03*	.15
	Environmental conditions	9.07	1.34	9.02	1.34	8.55	1.49	1.39	.26	.09	.03	.86	.00	2.77	.10	.09
	Health safety	9.75	0.63	9.15	1.40	9.43	0.87	4.60	.01*	.25	6.24	.01*	.17	4.01	.05*	.12

*M* = Mean; *SD* = standard deviation; *ɳ*_*p*_^*2*^ = partial eta-square; *p < .05; ** *p* < .001

Likewise, the linear model of analysis of variance indicated a higher score for the learning conditions in the virtual context compared with the outdoor context, with a large effect size that reached statistical significance (*M*_*outdoor*_ = 26.35; *M*_*indoor*_ = 27.00; *M*_*virtual*_ = 27. 95, *F*_*(1, 28)*_ = 3.81, *p* = 0.03, *η*_*p*_^*2*^ = 0.21). Bonferroni post-hoc tests revealed a significant difference in this dimension, with scores being higher in the outdoor context compared with the virtual context (*M*_*outdoor*_ = 26.35; *M*_*virtual*_ = 27. 95, *F*_*(1, 28)*_ = 6.52, *p* = 0.01, *η*_*p*_^*2*^ = 0.18). This same pattern of results was observed for health safety (*M*_*outdoor*_ = 9.43; *M*_*virtual*_ = 9.75; *F*_(1, 28)_ = 4.01, p = 0.05; *η*_*p*_^*2*^ = 0.12) and technical conditions (*M*_*outdoor*_ = 8.37; *M*_*virtual*_ = 9.13; F_(1, 28)_ = 5.20, *p* = 0.03; *η*_*p*_^*2*^ = 0.15). However, no statistically significant differences were found in relation to environmental conditions. Moreover, no statistically significant differences were found between the indoor and virtual contexts for the general factor of learning conditions. A statistically significant difference was only found in health security, with higher scores for the virtual condition (*M*_*indoor*_ = 9.15; *M*_*virtual*_ = 9.75; *F*_(1, 28)_ = 6.24, *p* = 0.01; *η*_*p*_^*2*^ = 0.17).

## Discussion

This study’s aim was to evaluate the impact of the MOTEMO-OUTDOOR programme, an innovative higher education teaching project based on active learning implemented during the COVID-19 pandemic. Starting from the seminars of a compulsory subject for the degree in psychology that in previous years had been delivered via a synchronous face-to-face indoor modality, the procedures, protocols, and teaching materials were redesigned to be taught both in this context and in informal learning environments—synchronous face-to-face outdoor and asynchronous online. With this adaptation, we aimed to provide the teaching–learning processes with flexibility at a time of extreme uncertainty caused by the pandemic, ensuring that these processes could be maintained regardless of the decisions made by the academic and health authorities. This innovation was based on the premise that any of the procedures should generate optimal and similar learning experiences, all of which would ensure good learning conditions for the students.

The results obtained for the overall sample showed that the outdoor environment was slightly superior to the indoor context regarding the learning experience, while the indoor context generally provided the best conditions for learning. However, it should be noted that the effect sizes of the differences found on the dimensions evaluated between both teaching–learning contexts, with partial eta-squared (*ɳ*_*p*_^*2*^) values between 2 and 7%, which are considered small (Richardson, 2010). Taking the indoor context as a reference, which is the one most similar to the teaching protocol employed prior to this teaching innovation project, scores in the different dimensions evaluated were very high (between 8.5 and 9.0 on a scale ranging from 0–10), suggesting that the range for improvement in these dimensions for the outdoor environment was limited because of a ceiling effect. Although the learning conditions were evaluated as being slightly lower in the outdoor format than the indoor format, this did not seem to have a negative influence on learning. The best results obtained in the outdoor context compared with indoor format in the hedonic dimension are in line with what has been reported in other studies such as the systematic review by Becker et al. (2017), which revealed an improvement in learning processes and motivation in various programs based on regular classes in outdoor settings. However, this comparison must be taken with caution, because this systematic review focused on the 5-year to 18-year age group.

Evidence concerning the flexibility of teaching–learning procedures developed in this project emerges from the results obtained for the subsample of students who experienced the three formats. In this group, the online format appeared to be a good alternative for learning when face-to-face interactions were not possible. Despite not having the social interactivity of the other two face-to-face formats, which partly explains the slightly-lower scores in the hedonic component, the learning experience in the virtual context did not seem to be significantly different from that of the face-to-face environments. This result is noteworthy considering that, among the elements that enhance student engagement, a key factor that was altered by the ‘educational lockdown’ was the tutor–student relationship. Within this relationship, teachers can enhance and promote the perspectives, interests, and visions of their students (Robinson, 2012). In this regard, Carrillo and Flores (2020) pointed to three main factors that impact teaching practices and promote the quality of the learning experience: (1) the presence of the teacher as a facilitator of learning; (2) social presence as an element of cohesion, and (3) cognitive presence in terms of reflective learning. Even though the tutoring process and the presence of teachers can partially remain intact in online classes (O'Dowd, 2019), there is a risk that the interruption of face-to-face teaching because of the pandemic negatively impacted the level of support that teachers could provide to their students. Nonetheless, the results of our study showed that the adaptations made to the online format overcame the effect of the reduced teacher–student interaction on learning. In fact, the virtual format presented some slight advantages regarding learning conditions, with significant favourable differences in perceived health security and technical conditions, which possibly compensated for the other less-favourable aspects of this teaching–learning format.

Taken together, analysis for the general sample and the subsample suggest that the multi-environment adaptation of the seminars based on active learning was successful from a student perspective. First, the overall scores in relation to the evaluated dimensions were very high regardless of the teaching and learning and, second, it appears that the differences in the learning experience between the three formats were minimised. Concerning the original teaching environment (indoor synchronous face-to-face), the online context seems to safeguard the learning experience and the outcomes expected by students. Perhaps this is attributable not only to the materials and procedures used in this format, but also to the fact that students were favoured by better technical and safety conditions for carrying out their activities, which might have compensated for the absence of a synchronous face-to-face interaction with the teacher. Moreover, the outdoor synchronous face-to-face model also seems to have ensured a learning experience comparable, if not superior, to the ‘default’ model (indoor synchronous face-to-face). Undoubtedly, the minimal differences in the outdoor context in terms of technical and environmental conditions were compensated by better perceived health safety and hedonic experience, because the lower experience of affective states of being threatened, together with a greater positive affect, might have favoured the learning processes. The trade-off between favourable and unfavourable factors could perhaps explain why, finally, the perception of learning and associated academic outcomes are the same in the indoor and outdoor formats.

These results seem to contradict most of the evidence reported regarding adaptation to online formats during the pandemic. Unlike other experiences, in which substituting the usual learning context for online teaching has been perceived negatively (Barton, 2020), the MOTEMO-OUTDOOR programme has been highly successful regardless of the teaching–learning format. In our opinion, this discrepancy could be explained by the fact that, while most of the transformations from a synchronous face-to-face format to the online format (synchronous or asynchronous) were reactive, sudden, and with little time for planning, the MOTEMO-OUTDOOR programme had enough time to be designed and implemented in planned and controlled manner, was based on a previous gamification experience, and always adopted an active learning approach. In summary, this teaching–learning model, designed to be implemented in higher education, seems to be highly resilient in situations of uncertainty and provides sufficient flexibility to ensure the optimal conditions for learning. In the words of Ali et al. (2020), this programme meets the conditions to be regarded as one of the “flexible and resilient education systems as we face unpredictable futures”.

Gender was included in this study as a possible moderating or confounding variable that deserves special attention because of its significance. A symmetrical pattern was observed between men and women regarding the assessment of the learning experience. Thus, while women valued the outdoor format more highly than the indoor format, the opposite was found for men. This same pattern was observed in two of the three dimensions (learning and hedonic experience). Regarding the learning conditions, the results are inconclusive because differences between men and women are consistent with those obtained for the learning experience factor (i.e., men are more critical than women regarding the technical and environmental conditions of learning, especially in the outdoor context), although these differences do not reach statistical significance probably because of the combination of a small sample with a small effect size. However, the covariance analysis conducted to examine the effect of gender on the learning experience, in which controlling for the dimensions of learning conditions eliminates the effect of gender, suggests that it is precisely the more negative perception of such conditions by male students that would lead them to rate their learning experience more negatively. We can rule out an explanation for this effect in terms of perceived health security, because men showed higher scores on this construct. In any case, this gender difference is in line with previous evidence that has consistently shown how women have a greater perception of threat in any context, which might be related both to gender stereotypes and/or gender differences in dispositional variables (Lippa, 2010). This teaching innovation project in higher education has been carried out within the context of psychology, a strongly-feminised health sciences degree. Thus, a gender perspective should be considered when implementing teaching–learning procedures such as the one presented here, especially in university degrees with an uneven gender distribution. Thus, we should consider the generalizability of our findings. Aside from the gender issue just described, the age of the participant was practically a constant, with most of them in the age range of 18 to 20 years. This makes it impossible to carry out a bias-free analysis of this variable that would provide evidence to support its implementation at other educational levels.

The results obtained in this study are robust. Although not having been obtained through objective measurements, the results could present a series of limitations. In the first place, it is worth mentioning that an assessment of the real evaluative impact was not carried out because the design of the study itself prevented it. The purpose of this study was not to conduct a verification of new methods in the event of their need for implementation, as the need for implementation already existed at the time of the study. It seemed unethical to carry out a design between groups, with an intervention group and a control group, because all students should benefit. Therefore, we chose a within-subject design (with the advantages that it brings in relation to the sample power because each person is a control of herself). In this way, the assignment of the performance context did not fall on the student, but on the seminar. Likewise, there was a third context, the virtual synchronous, over which the research team did not have assignment control. The fact that a student carried out one or more of the seminars in virtual format was a consequence of the application of the COVID protocol in force at that time in the university. This is why the number of people who finally met the eligibility criteria to be included in the study and also had experience of a virtual seminar comprised a small subsample that was analysed separately. Therefore, there are reservations regarding the conclusions derived from the results mostly because of the modest size of the sample.

Another limitation of this study was the focus on student opinion measures as opposed to external criteria such as real performance indicators, which were not possible for two reasons. First, the within-subject nature of the study, which included students of the whole population who required the programme, made it impossible to verify whether the assignment to different teaching contexts influenced academic performance. Second, the design of the activity for each for the seminars submitted to this triple adaptation of the format made it possible for the student to obtain feedback and opportunities to rectify their responses that virtually ensured perfect performance. Therefore, performance was almost a constant for each learning environment and student, which ruled it out as a valid indicator. However, in the future, if the exogenous conditions and the intrinsic conditions of the teaching–learning process allow, the inclusion of performance indicators should be considered in relation to the intervention carried out, along with the rest of the indicators evaluated.

Moreover, we decided to use pseudo-randomisation—and not strict randomisation—for two reasons. First, this decision was based on previous teaching organisation protocols in the faculty (established by the teacher of each group). Second, a homogeneous distribution of a discrete number of sessions (*k* = 78) between levels (group of students, teacher, and seminar) was required. A strict randomisation protocol most likely would have generated a heterogeneous distribution of sessions between the three allocation levels and produced an effect of confounding variables. The study design was indeed robust, because we included this pseudo-randomisation and used counterbalancing to control for another possible confounding factor (i.e., the order in which the seminars were held). Moreover, none of these confounding variables were predictors of the outcome variables and therefore they did not have to be incorporated as adjustment variables in any of the statistical models used in the study.

To conclude, our findings suggest that optimal conditions have been created for implementing the programme in the three environments assessed (indoor, outdoor, and online). The outdoor environment has allowed for promotion of a good learning experience, probably because of the impact of the green spaces on attentional processes and the mobilization of positive emotions. The benefits attributed to outdoor education refer in a very broad sense to a set of educational practices in nature and go far beyond the simple geographical transfer and adaptation of educational procedures originally designed for formal contexts in spaces inside buildings (i.e. educational institutions expressly designed for student–teacher interaction). In this set of educational practices, supposedly initiated before the Neolithic age, reference is also made to the use of active interaction with nature itself for the development of physical and cognitive skills and for acquiring knowledge of that nature (Parker, 2022). In terms of learning objectives and procedures, all this goes beyond the experience developed with the MOTEMO-OUTDOOR programme, because it aimed to maintain the teaching–learning process during the pandemic, which is an objective that appears to have been achieved according to the results obtained.

It is possible that the present results, in line with what was pointed out in the Broaden-and-Build theory (Fredrickson, 2004), could be a consequence of the activation of positive emotions during learning, while we might also speculate whether this effect is attributable to the cognitive benefits of natural spaces, an idea proposed by Kaplan in his Attention Restoration Theory (1995). Similarly, the online context has provided better learning conditions, which seems to have compensated for the reduction in student–teacher interaction to produce a learning experience comparable to the classic format (indoor). Moreover, our results confirm a clear relationship between the learning conditions and the learning experience. Therefore, the success of the programme does not only rely on the reshaping of the dynamics of student–teacher interaction and teaching materials, but also on the choice of spaces and the strict application of the decision protocol (see supplementary material SM4) of holding sessions in an outdoor context, which is essentially based on weather conditions. In this regard, it is important to remember that this project was conducted in an area with a Mediterranean climate and during the spring season, which have greatly facilitated both its implementation and the subsequent positive outcomes. The sociodemographic status of the students who participated in this study must also be considered. In particular, it is important to note that all the students had minimum technical resources essential for optimal use of the designed seminars. This is relevant because any attempt to implement online teaching must overcome the inequalities between students in terms of accessing technologies, a divide that can negatively affect the learning opportunities of disadvantaged students (Beaunoyer et al., 2020; Kapasia et al., 2020). Indeed, some studies have highlighted the fundamental importance of providing adequate conditions in home learning environments to promote online learning sessions characterized by high levels of motivation and well-being and low levels of stress (Aschenberger et al., 2022).

## Supplementary Information

Below is the link to the electronic supplementary material.

Supplementary file1 (DOCX 14 kb)

Supplementary file2 (DOCX 1119 kb)

Supplementary file3 (DOCX 12 kb)

Supplementary file4 (DOCX 17 kb)

Supplementary file5 (DOCX 12 kb)

Supplementary file6 (DOCX 12 kb)

Supplementary file7 (DOCX 12 kb)
