# Supplementary material for: MOTEMO-OUTDOOR: ensuring learning and health security during the COVID-19 pandemic through outdoor and online environments in higher education
Source: Learn Environ Res. 2023 Feb 9:1–19. Online ahead of print. doi: 10.1007/s10984-023-09456-y (PMC9909139; doi:10.1007/s10984-023-09456-y)
Supplement: Supplementary file 1 — Supplementary file1 (DOCX 14 kb) [file 10984_2023_9456_MOESM1_ESM.docx]

**Supplementary Material 1 (SM1)**

**MOTEMO-OUTDOOR program: Teaching, context, and resources adaptation**

The adaptations for the MOTEMO-OUTDOOR programme were based on the previous format for carrying out the seminars in classroom face-to-face synchronous format, which has been applied in a standardised way for the four seminars that were implemented in the context of the study plan of the degree in psychology of the UAB in force since the adaptation to the Bologna plan of the European Space of Higher Education from the 2010-11 academic year. Two of these seminars (SEM1 and SEM3) are dedicated to analysing real cases, and two of them (SEM2 and SEM4) are dedicated to reviewing concepts and resolving doubts about the theoretical contents of the subject. This previous format was always taught synchronously in classrooms adapted for seminar teaching in groups of 20 students led by a professor. The dynamics of each seminar session consisted of a discussion by teams of 4 to 5 students. The professor presented each case assisted by the projection of a multimedia material designed in PowerPoint, gave a time for internal discussion, the students provided an oral and justified response to the analysis, and finally the professor proposed an argued resolution of the case, with resolution of doubts. An adaptation of multimedia materials, dynamics and equipment was designed for the MOTEMO-OUTDOOR programme in order to be able to carry out the seminars both to the outdoor synchronous face-to-face format and to the asynchronous virtual context, trying to homogenise both learning contexts with the format as much as possible with the original format (indoor synchronous face-to-face) in order to minimise the possible differences in the learning processes between all of them due to the intrinsic differences between the different contexts.

In this way, an adaptation of the multimedia teaching materials was made (see supplementary material SM1), which were embedded into forms designed with Google Forms^®^:

Links to teaching materials:

Seminar 1: <https://forms.gle/3bUgaHZh8RZNedod7>

Seminar 2: <https://forms.gle/Pwsskx4AdgEAFFWL7>

Seminar 3: <https://forms.gle/yMyTgnprrGbRcjFX8>

Seminar 4: <https://forms.gle/3zMaiAw3QxH9aF9b6>

These forms presented the same cases that were previously contained in the multimedia teaching material projected by the professor, which includes videos, text, infographics, and photographs. The case raised orally by the professor was replaced by a statement accompanied by various response options (between 2 and 6). All forms provided feedback on results and the opportunity to modify answers. To guarantee homogeneity, access to the teaching material was always made from links available in the virtual classroom, regardless of the context (indoor, outdoor or virtual) in which the student carried out the activity.

Additionally, to be able to carry out the seminars in the outdoor synchronous face-to-face context in green spaces of the university campus, three actions were carried out.

1. A 60 x 80 cm plastic roll-up mat was designed with sufficient hardness to guarantee comfort during the 100-min duration of the seminars sitting on an uneven surface (the grass), and with a sufficient size to be able to sit down and place the electronic device used to follow the sessions.
2. A search and selection of optimal outdoor spaces was carried out. The eligibility criteria were: (1) maximum distance of 200m from the entrances to the faculty, (2) green area, and (3) good wi-fi coverage, (4) low mobility in the surroundings, and (5) a sustained average level of environmental noise under 60dB. A total of 5 spaces that met the eligibility criteria were located, among which the members of the teaching team responsible for teaching seminars (AS, CM and NC) prioritised two, space A and space E (see Figure 1), who were finally employed throughout the implementation of the entire program.
3. A logistics procedure related to communication, material resources and displacements was designed and informed to the students (see supplementary material SM2).

An Extech 407736 Sound Level Meter was used to analyse the acoustic conditions of the outdoor spaces to be selected for the programme. This analysis was done according to the eligibility conditions that were used to select the possible spaces (see the next section).

To evaluate the adequacy of the weather conditions for holding the seminar sessions in the outdoor context, data by the Catalan Meteorological Service (2021) from the Sant Cugat del Vallès station were obtained in real time.

For each session, the professor recorded the following metadata, some of them in redundancy to those provided by the students for data verification and error correction: the seminar given, the delivery context (indoor vs outdoor), and the teacher who taught it.
