# Supplementary figures and images for: MOTEMO-OUTDOOR: ensuring learning and health security during the COVID-19 pandemic through outdoor and online environments in higher education
Source: Learn Environ Res. 2023 Feb 9:1–19. Online ahead of print. doi: 10.1007/s10984-023-09456-y (PMC9909139; doi:10.1007/s10984-023-09456-y)

**Supplementary material 2 (SM2)**

Logistic document shared with the students:

**
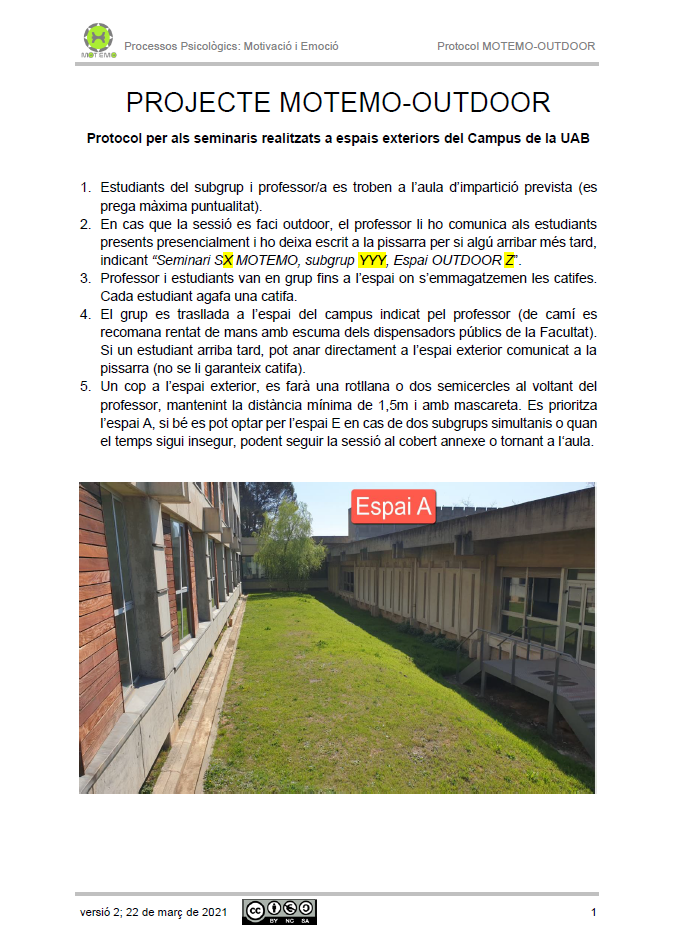
**

**
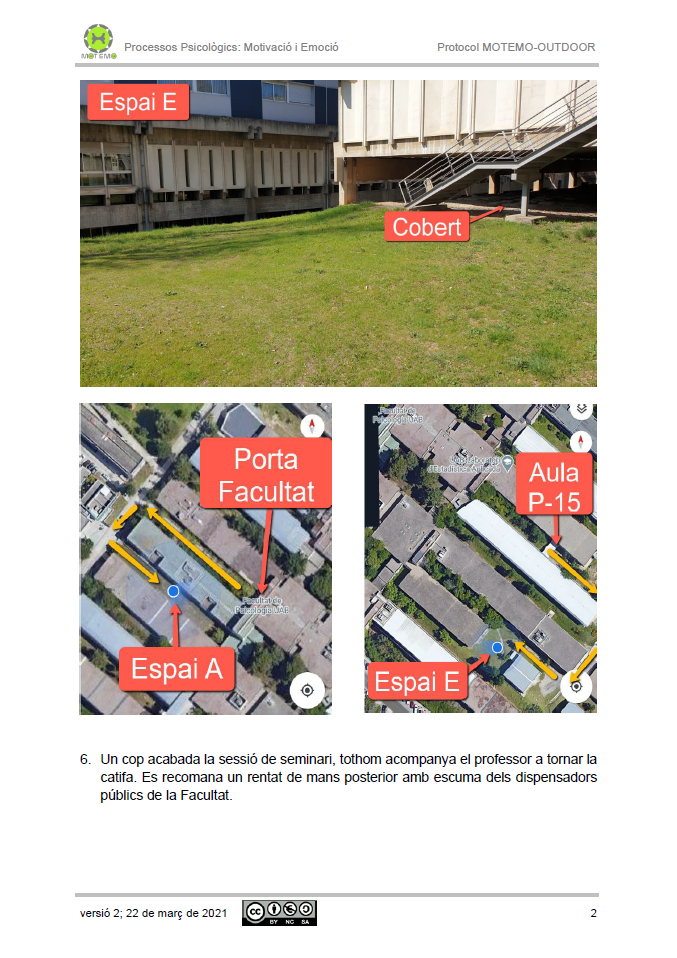
**

Supplement: Supplementary file 2 — Supplementary file2 (DOCX 1119 kb) [file 10984_2023_9456_MOESM2_ESM.docx]
