# Supplementary material for: MOTEMO-OUTDOOR: ensuring learning and health security during the COVID-19 pandemic through outdoor and online environments in higher education
Source: Learn Environ Res. 2023 Feb 9:1–19. Online ahead of print. doi: 10.1007/s10984-023-09456-y (PMC9909139; doi:10.1007/s10984-023-09456-y)
Supplement: Supplementary file 3 — Supplementary file3 (DOCX 12 kb) [file 10984_2023_9456_MOESM3_ESM.docx]

**Supplementary Material 3 (SM3)**

Session assignments by professor as scheduled in the faculty programming:

|  |  |  |  |  |
| --- | --- | --- | --- | --- |
|  | **Teacher Assignment** | | | |
| **Students' Group** | **Seminar 1** | **Seminar 2** | **Seminar 3** | **Seminar 4** |
| **1** | CM | AS | AS | AS |
| **2** | NC | NC | NC | NC |
| **3** | CM | CM | CM | NC |
| **4** | AS | AS | AS | AS |
| **5** | CM | CM | CM | NC |
| **6** | AS | AS | AS | AS |
| **7** | NC | AS | AS | NC |
| **8** | AS | AS | AS | AS |
| **9** | CM | CM | CM | CM |
| **10** | NC | NC | NC | NC |
| **11** | CM | CM | CM | CM |
| **12** | NC | NC | NC | NC |
| **13** | CM | CM | CM | CM |
| **14** | NC | NC | NC | NC |
| **15** | CM | CM | CM | CM |
| **16** | NC | NC | NC | NC |
| **17** | NC | NC | NC | NC |
| **18** | CM | CM | CM | CM |
| **19** | CM | NC | CM | NC |
|  |  |  |  |  |
|  | **AS** | **CM** | **NC** | **TOTAL** |
| **Sessions by professor** | 17 | 29 | 30 | 76 |
|  |  |  |  |  |
