# Supplementary material for: MOTEMO-OUTDOOR: ensuring learning and health security during the COVID-19 pandemic through outdoor and online environments in higher education
Source: Learn Environ Res. 2023 Feb 9:1–19. Online ahead of print. doi: 10.1007/s10984-023-09456-y (PMC9909139; doi:10.1007/s10984-023-09456-y)
Supplement: Supplementary file 4 — Supplementary file4 (DOCX 17 kb) [file 10984_2023_9456_MOESM4_ESM.docx]

**Supplementary Material 4 (SM4)**

Context assignment of all the seminar sessions, and their absolute and relative distribution by seminar, student, and professor:

|  |  |  |  |  |  |  |  |
| --- | --- | --- | --- | --- | --- | --- | --- |
|  |  | **context assignment** | | | | **Outdoor by student** | |
| **Students Group** | | **Seminar 1** | **Seminar 2** | **Seminar 3** | **Seminar 4** | **k** | **%** |
| **1** | | indoor | indoor (r)* | outdoor | outdoor (r)* | 2 | 50,0 |
| **2** | | indoor | outdoor | indoor | outdoor | 2 | 50,0 |
| **3** | | outdoor | indoor | outdoor | indoor | 2 | 50,0 |
| **4** | | outdoor | outdoor | indoor | indoor | 2 | 50,0 |
| **5** | | outdoor | indoor | indoor | outdoor | 2 | 50,0 |
| **6** | | indoor | outdoor | indoor | outdoor | 2 | 50,0 |
| **7** | | outdoor | outdoor | indoor | indoor | 2 | 50,0 |
| **8** | | indoor | outdoor | outdoor | indoor | 2 | 50,0 |
| **9** | | outdoor | outdoor | indoor | indoor | 2 | 50,0 |
| **10** | | outdoor | outdoor | indoor | indoor | 2 | 50,0 |
| **11** | | indoor | outdoor | outdoor | indoor | 2 | 50,0 |
| **12** | | outdoor | indoor | outdoor | indoor | 2 | 50,0 |
| **13** | | outdoor | indoor | indoor | outdoor | 2 | 50,0 |
| **14** | | outdoor | outdoor | indoor | indoor | 2 | 50,0 |
| **15** | | outdoor | indoor | indoor | outdoor | 2 | 50,0 |
| **16** | | indoor | indoor | outdoor | outdoor | 2 | 50,0 |
| **17** | | indoor | indoor | outdoor | outdoor | 2 | 50,0 |
| **18** | | outdoor | indoor | outdoor | indoor | 2 | 50,0 |
| **19** | | indoor | indoor | outdoor | outdoor | 2 | 50,0 |
| **Outdoor by session** | **k** | 11 | 9 | 9 | 9 | 38 |  |
|  | **%** | 57,9 | 47,4 | 47,4 | 47,4 |  | 50,0 |
| *(r) = Rescheduled by permutation of sessions format | | | | | | | |
|  |  |  |  |  |  |  |  |
|  |  | **AS** | **CM** | **NC** | **TOTAL** |  |  |
| **Outdoor by professor** | **k** | 9 | 14 | 15 | 38 |  |  |
|  | **%** | 52,9 | 48,3 | 50,0 | 50,0 |  |  |

**Decision protocol**

In anticipation of adverse weather conditions that would require rescheduling the context of a significant number of sessions, and in order to keep the number of indoor and outdoor sessions scheduled for each group and globally constant, it was decided that (1) the *k* = 38 sessions scheduled in outdoor context (50% of the total), would initially be scheduled unevenly following a decreasing number seminar by seminar (SEM1: outdoor = 11, SEM2: outdoor = 10, SEM3: outdoor = 9; SEM4: outdoor = 8) to, if necessary (2) reschedule such sessions interchanging the context with another of the same group of students. The rescheduling weather conditions were as follows: (1) temperature below 15ºC (in any case), or (2) temperature below 20ºC combined with cloudy skies, or wind speeds greater than 20 km/h. These conditions were chosen (1) considering the climatology of the area (Weatherspark, 2021), (2) avoiding a strong discrepancy with indoor environmental conditions to maintain high comparability with outdoor sessions, and (3) avoiding a high number of sessions to reschedule. According to real-time verification of data from the nearest official weather station (Servei Meteorològic de Catalunya, 2021), only one out of the 38 sessions (2.6% of the total) did not meet the expected weather conditions, so it was rescheduled. Instead, to keep the number of seminars held in both outdoor and indoor formats constant, another session of the affected group of students was rescheduled to an outdoor format.

Servei Meteorològic de Catalunya (2021). Dades de l’estació automàtica de Sant Cugat del Vallès - CAR [Data from the Sant Cugat del Vallès - CAR automatic station]. Retrieved from April 01, 2021 to June 10, 2021 from https://www.meteo.cat/observacions/xema/dades?codi=XV&dia=2021-04-01T00:00Z

Weatherspark (2021). Climate and Average Weather Year Round in Cerdanyola del Vallès (Spain). Retrieved April 01, 2021 from https://weatherspark.com/y/47247/Average-Weather-in-Cerdanyola-del-Vall%C3%A8s-Spain-Year-Round
