# Supplementary material for: MOTEMO-OUTDOOR: ensuring learning and health security during the COVID-19 pandemic through outdoor and online environments in higher education
Source: Learn Environ Res. 2023 Feb 9:1–19. Online ahead of print. doi: 10.1007/s10984-023-09456-y (PMC9909139; doi:10.1007/s10984-023-09456-y)
Supplement: Supplementary file 5 — Supplementary file5 (DOCX 12 kb) [file 10984_2023_9456_MOESM5_ESM.docx]

**Supplementary material SM5.** Correlation matrix for outdoor environment

| Factor | Dimension | Learning experience | | | |  | Learning conditions | | |
| --- | --- | --- | --- | --- | --- | --- | --- | --- | --- |
|  |  | Total | Learning | Evaluation | Hedonic |  | Total | Technical | Environmental |
| Learning experience | Total |  |  |  |  |  |  |  |  |
|  | Learning | .85** |  |  |  |  |  |  |  |
|  | Evaluation | .83** | .65** |  |  |  |  |  |  |
|  | Hedonic | .81** | .52** | .44** |  |  |  |  |  |
| Learning Conditions | Total | .46** | .37** | .29** | .47** |  |  |  |  |
|  | Technical | .27** | .28** | .20** | .23** |  | .79** |  |  |
|  | Environmental | .39** | .27** | .21** | .47** |  | .79** | .34** |  |
|  | Safety | .38** | .36** | .26** | .33** |  | .59** | .31** | .27** |
| * The correlation is significant at the .05 level (two-tailed). | | | |  |  |  |  |  |  |
| ** The correlation is significant at the .01 level (two-tailed). | | | |  |  |  |  |  |  |
|  |  |  |  |  |  |  |  |  |  |
