# Supplementary material for: MOTEMO-OUTDOOR: ensuring learning and health security during the COVID-19 pandemic through outdoor and online environments in higher education
Source: Learn Environ Res. 2023 Feb 9:1–19. Online ahead of print. doi: 10.1007/s10984-023-09456-y (PMC9909139; doi:10.1007/s10984-023-09456-y)
Supplement: Supplementary file 6 — Supplementary file6 (DOCX 12 kb) [file 10984_2023_9456_MOESM6_ESM.docx]

**Supplementary material SM6.** Correlation matrix for indoor environment

| Factor | Dimension | Learning experience | | | |  | Learning onditions | | |
| --- | --- | --- | --- | --- | --- | --- | --- | --- | --- |
|  |  | Total | Learning | Evaluation | Hedonic |  | Total | Technical | Environmental |
| Learning experience | Total |  |  |  |  |  |  |  |  |
|  | Learning | .88** |  |  |  |  |  |  |  |
|  | Evaluation | .86** | .71** |  |  |  |  |  |  |
|  | Hedonic | .83** | .60** | .52** |  |  |  |  |  |
| Learning conditions | Total | .37** | .37** | .29** | .30** |  |  |  |  |
|  | Technical | .27** | .23** | .22** | .14* |  | .81** |  |  |
|  | Environmental | .28** | .28** | .18** | .27** |  | .80** | .40** |  |
|  | Safety | .40** | .39** | .30** | .36** |  | .71** | .36** | .46** |
| * The correlation is significant at the .05 level (two-tailed). | | | | |  |  |  |  |  |
| ** The correlation is significant at the .01 level (two-tailed). | | | | |  |  |  |  |  |
|  |  |  |  |  |  |  |  |  |  |
