# Supplementary material for: MOTEMO-OUTDOOR: ensuring learning and health security during the COVID-19 pandemic through outdoor and online environments in higher education
Source: Learn Environ Res. 2023 Feb 9:1–19. Online ahead of print. doi: 10.1007/s10984-023-09456-y (PMC9909139; doi:10.1007/s10984-023-09456-y)
Supplement: Supplementary file 7 — Supplementary file7 (DOCX 12 kb) [file 10984_2023_9456_MOESM7_ESM.docx]

**Supplementary material SM7.** Correlation matrix for online environment

| Factor | Dimension | Learning experience | | | |  | Learning conditions | | |
| --- | --- | --- | --- | --- | --- | --- | --- | --- | --- |
|  |  | Total | Learning | Evaluation | Hedonic |  | Total | Technical | Environmental |
| Learning experience | Total |  |  |  |  |  |  |  |  |
|  | Learning | .94** |  |  |  |  |  |  |  |
|  | Evaluation | .92** | .81** |  |  |  |  |  |  |
|  | Hedonic | .89** | .68** | .70** |  |  |  |  |  |
| Learning conditions | Total | .60** | .57** | .60** | .42** |  |  |  |  |
|  | Technical | .60** | .58** | .62** | .47* |  | .90** |  |  |
|  | Environmental | .55** | .56** | .52** | .22** |  | .89** | .68** |  |
|  | Safety | .29* | .24 | .33** | .24 |  | .73** | .50** | .50** |
| * The correlation is significant at the .05 level (two-tailed). | | | | |  |  |  |  |  |
| ** The correlation is significant at the .01 level (two-tailed). | | | | |  |  |  |  |  |
|  |  |  |  |  |  |  |  |  |  |
